# Supplementary material for: Cdk5 and GSK3β inhibit fast endophilin-mediated endocytosis
Source: Nat Commun. 2021 Apr 23;12:2424. doi: 10.1038/s41467-021-22603-4 (PMC8065113; doi:10.1038/s41467-021-22603-4)
Supplement: Supplementary file 1 — Supplementary Information [file 41467_2021_22603_MOESM1_ESM.pdf]

SUPPLEMENTARY INFORMATION

Cdk5 and GSK3β inhibit Fast Endophilin-Mediated Endocytosis

Antonio P. A. Ferreira[1,2,*], Alessandra Casamento[1,*], Sara Carrillo Roas[1], Els F. Halff[3,4], James Panambalana[1], Shaan Subramaniam[1,5], Kira Schützenhofer[1], Laura Chan Wah Hak[1,6], Kieran McGourty[1,7], Konstantinos Thalassinos[1], Josef T. Kittler[3], Denis Martinvalet[8] and Emmanuel Boucrot[1,5,$]

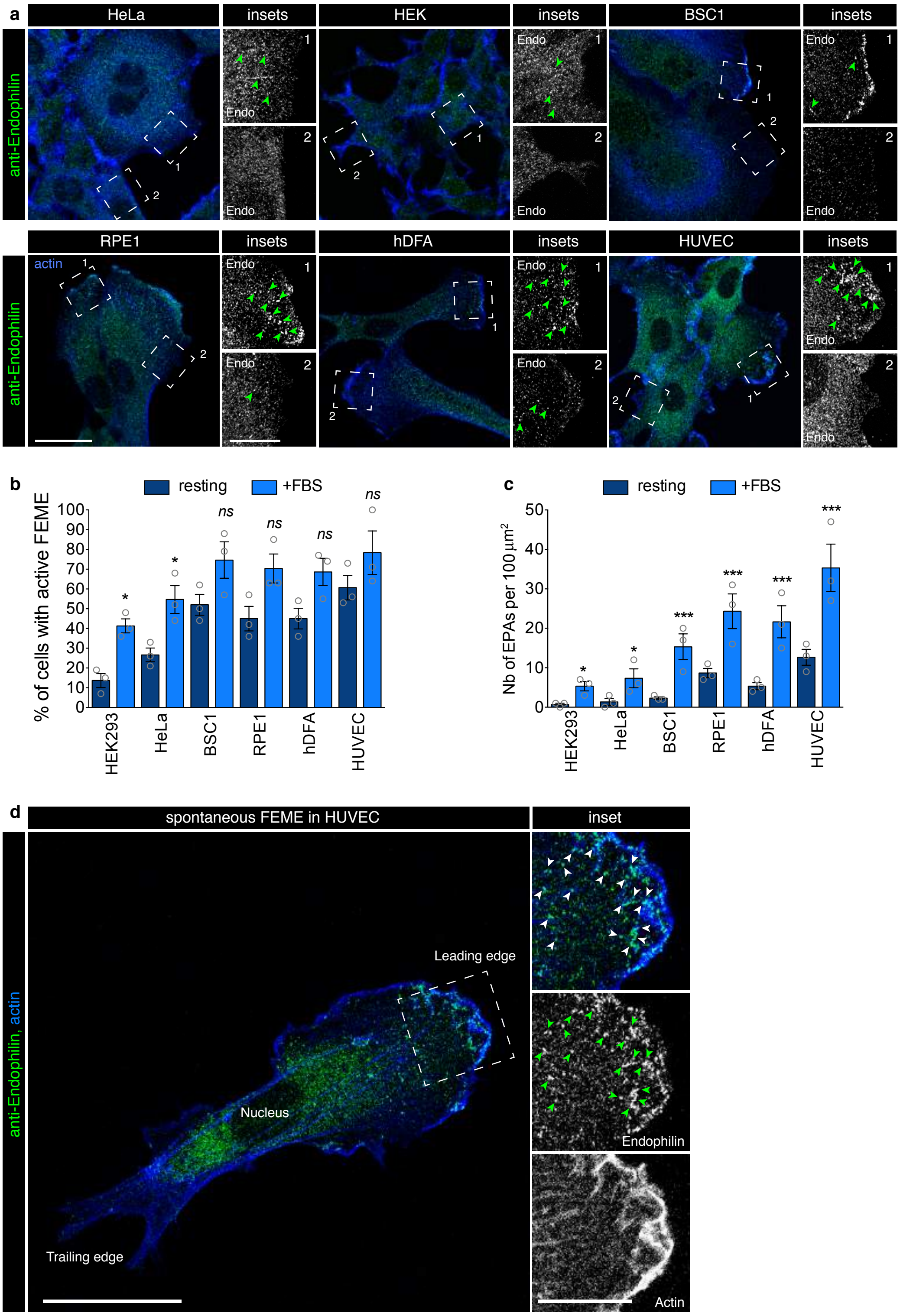

Supplementary Figure 1 *(previous page)***. Related to Figures 1 and 2. a**, Spontaneous FEME carrier formation in HeLa, HEK293, BSC1, RPE1, *human* primary dermal fibroblasts (hDFA) or HUVEC cells grown in their respective complete culture media (see Methods). Arrowheads point at FEME carriers. Scale bars, 20μm (main) and 10μm (inset). **b,** Histograms show the mean ± SEM of the percentage of resting or stimulated (+10% FBS) cells displaying active FEME from 3 independent biological experiments (n>50 cells per condition). Statistical analysis was performed by two-way ANOVA; *ns*, non significant; *, *P*<0.05. **c,** Histograms show the mean ± SEM of the number of FEME carriers in resting or stimulated (+10% FBS) cells from 3 independent biological experiments (n>150 EPAs per condition). Statistical analysis was performed by two-way ANOVA; *, *P*<0.05, ***, *P*<0.001. **d,** Example of spontaneous FEME in migrating HUVEC cells. Arrowheads point at FEME carriers (Endophilin-positive assemblies; tubules and vesicles) detected by confocal microscopy in the cytoplasm. Scale bar, 30μm (main) and 15μm (inset).

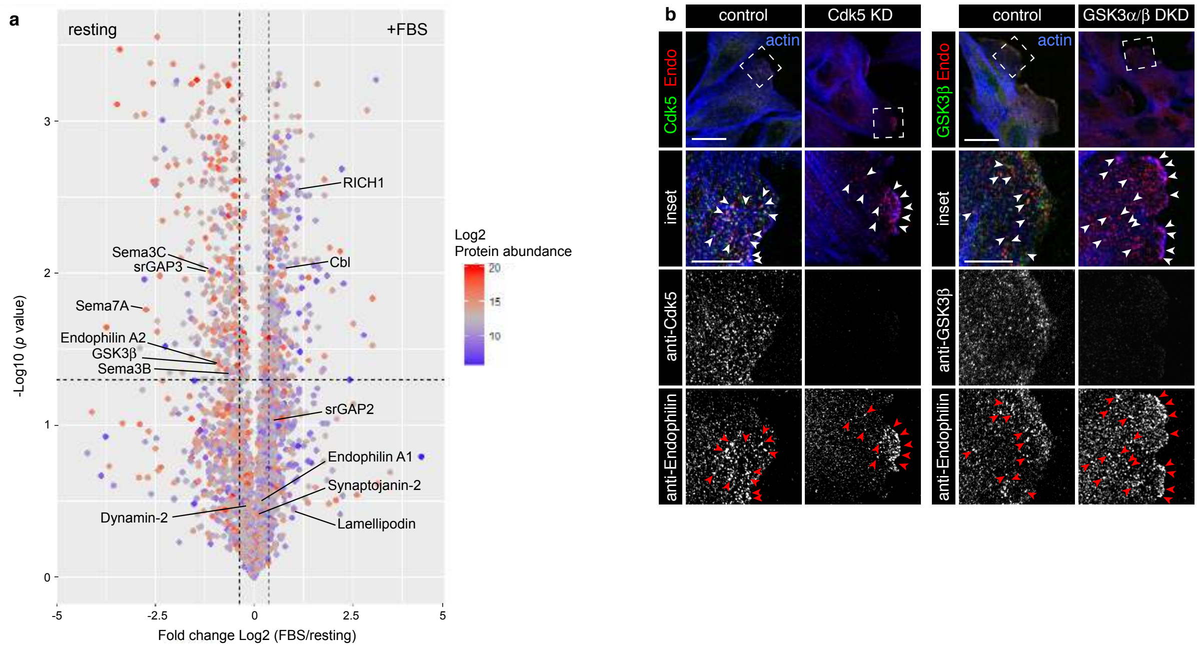

Supplementary Figure 2. Related to Figure 3. a, Volcano plot of -Log10 of *p* values versus Log2 of fold changes in proteins levels; Log2 of protein abundances are shown as heat map representation (blue=0, red=20) of proteins detected by mass spectrometry from fractions immunoprecipitated with anti-Endophilin antibodies. Cells were stimulated with additional 10% serum (FBS) or not (resting) prior to extraction and co-immunoprecipitation. Proteins relevant to this study were annotated. The full list of the proteins detected is provided in the Source Data file **b**, Confocal images showing the levels of Endophilin and Cdk5 or GSK3α/β in cells depleted of Cdk5 (CDK5 KD) or GSK3α/β (GSK3α/β DKD), respectively. Arrowheads point at FEME carriers. Scale bars, 20μm (main) and 10μm (inset).

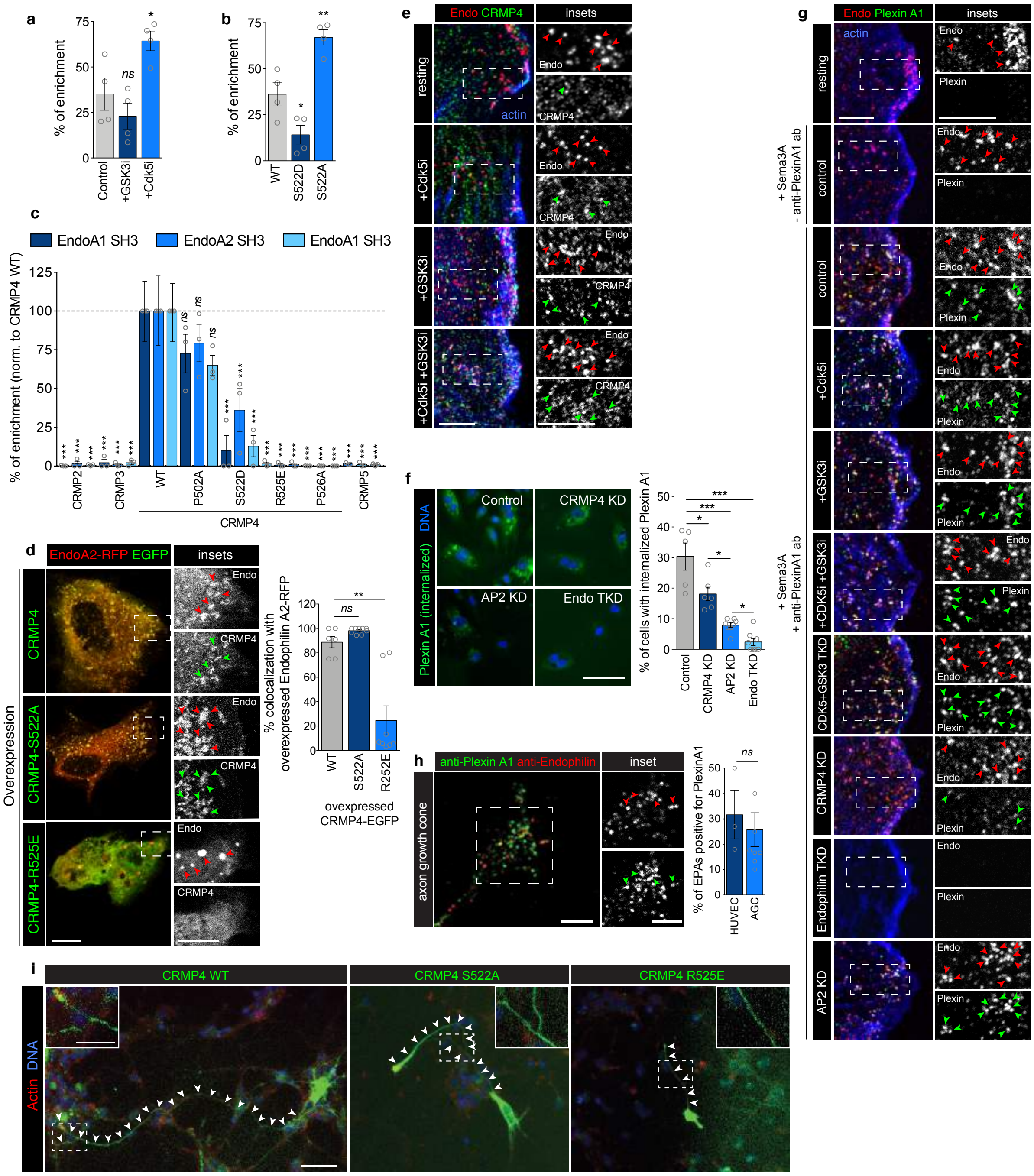
a
% of enrichment
Control
+GSK3i
+Cdk5i
b
WT
S522D
S522A
c
EndoA1 SH3
EndoA2 SH3
EndoA1 SH3
% of enrichment (norm. to CRMP4 WT)
CRMP2
CRMP3
WT
P502A
S522D
R525E
P526A
CRMP5
CRMP4
d
EndoA2-RFP EGFP
insets
Overexpression
CRMP4
CRMP4-S522A
CRMP4-R525E
Endo
% colocalization with overexpressed Endophilin A2-RFP
R252E
ovexpressed CRMP4-EGFP
e
Endo CRMP4
resting
+Cdk5i
+GSK3i
+Cdk5i +GSK3i
actin
f
Plexin A1 (internalized) DNA
Control
CRMP4 KD
AP2 KD
Endo TKD
% of cells with internalized Plexin A1
g
Endo Plexin A1
+ Sema3A - anti-PlexinA1 ab
+ Sema3A + anti-PlexinA1 ab
control
+CDK5i +GSK3i
CDK5+GSK3 TKD
Endophilin TKD
Plexin
h
anti-Plexin A1 anti-Endophilin
inset
axon growth cone
% of EPAs positive for PlexinA1
HUVEC
AGC
i
CRMP4 WT
CRMP4 S522A
CRMP4 R525E
Actin DNA

Supplementary Figure 3. *(previous page)* **Related to Figure 5. a**, Quantification of experiments illustrated in Figure 5b. Histograms show the mean ± SEM from 3 independent biological experiments. Statistical analysis was performed by one-way ANOVA; *ns*, non significant; *, $P<0.05$. **b**, Quantification of experiments illustrated on Figure 5c. Histograms show the mean ± SEM from 3 independent biological experiments. Statistical analysis was performed by one-way ANOVA; *, $P<0.05$, **, $P<0.01$. **c**, Quantification of experiments illustrated on Figure 5d. Histograms show the mean ± SEM from 3 independent biological experiments. Statistical analysis was performed by two-way ANOVA; *ns*, non significant; ***, $P<0.001$. **d**, Recruitment of the indicated overexpressed EGFP-tagged CRMP4 constructs onto structures formed by overexpressed Endophilin-A2-RFP. Arrowheads point at overexpressed Endophilin-RFP structures. Scale bar, 20μm (main) and 10μm (inset). Histograms show the mean ± SEM from 3 independent biological experiments ($n=15$ cells per condition). Statistical analysis was performed by one-way ANOVA; *ns*, non significant; **, $P<0.01$. **e**, Related to Figure 5f: whole dataset (note that some images are similar, as Figure 5f only shows a subset of conditions tested). Recruitment of endogenous CRMP4 onto FEME carriers in HUVEC cells treated for 10min with 5μM Dinaciclib (Cdk5i) and/or CHIR-99021 (GSK3i), or left untreated (resting). Arrowheads point at FEME carriers. Scale bars, 5μm. **f**, Internalized Plexin A1 (whole uptake, FEME plus other pathways) in control cells or cells depleted for CRMP4, AP2 or endophilin A1, A2 and A3 (Endo TKD), upon stimulation with 20nM Semaphorin 3A with 10μg/mL anti-PlexinA1 antibodies (recognizing the ectodomain of PlexinA1) for 20min. Unbound and cell surface bound anti-PlexinA1 antibodies were removed prior to fixation. Scale bar, 100μm. Histograms show the mean ± SEM from 3 independent biological experiments ($n>100$ cells per condition). Statistical analysis was performed by one-way ANOVA; *, $P<0.05$, ***, $P<0.001$. **g**, Related to Figure 5g: whole dataset (note that some images are similar, as Figure 5g shows a subset of the conditions tested). Endogenous Plexin A1 uptake into FEME carriers in HUVEC cells depleted of Endophilin A1, A2 and A3 (Endophilin TKD), Cdk5 and GSK3α and β (CDK5+GSK3α/β TKD), CRMP4 or AP2 or pre-treated with Cdk5i and/or GSK3i for 5min. Cells were stimulated by 20nM Semaphorin 3A (Sema3A) for 5min in presence of 10μg/mL anti-PlexinA1 antibodies (recognizing the ectodomain of PlexinA1) or left untreated (resting). Arrowheads point at FEME carriers. Scale bars, 5μm. **h**, Colocalization of endogenous Plexin A1 (green) and Endophilin (red) at axon growth cones (AGC) of *mouse* hippocampal neurons. Arrowheads point at endogenous Plexin A1 in FEME carriers (Endophilin-positive assemblies, EPAs). Scale bars, 5μm. Histograms show the mean ± SEM from 3 independent biological experiments ($n=90$ cells per condition). Statistical analysis was performed by one-way ANOVA; *ns*, non significant. **i**, *Mouse* hippocampal neurons expressing EGFP-CRMP4 wild type (WT), EGFP-CRMP4 S522A or EGFP-CRMP4 R525E mutants and stained for actin (red) and DNA (blue). Arrowheads highlight axons. Insets highlight axon branching (or lack thereof). The images are representative of 3 independent biological experiments. Scale bar, 80μm (main) and 40μm (inset).

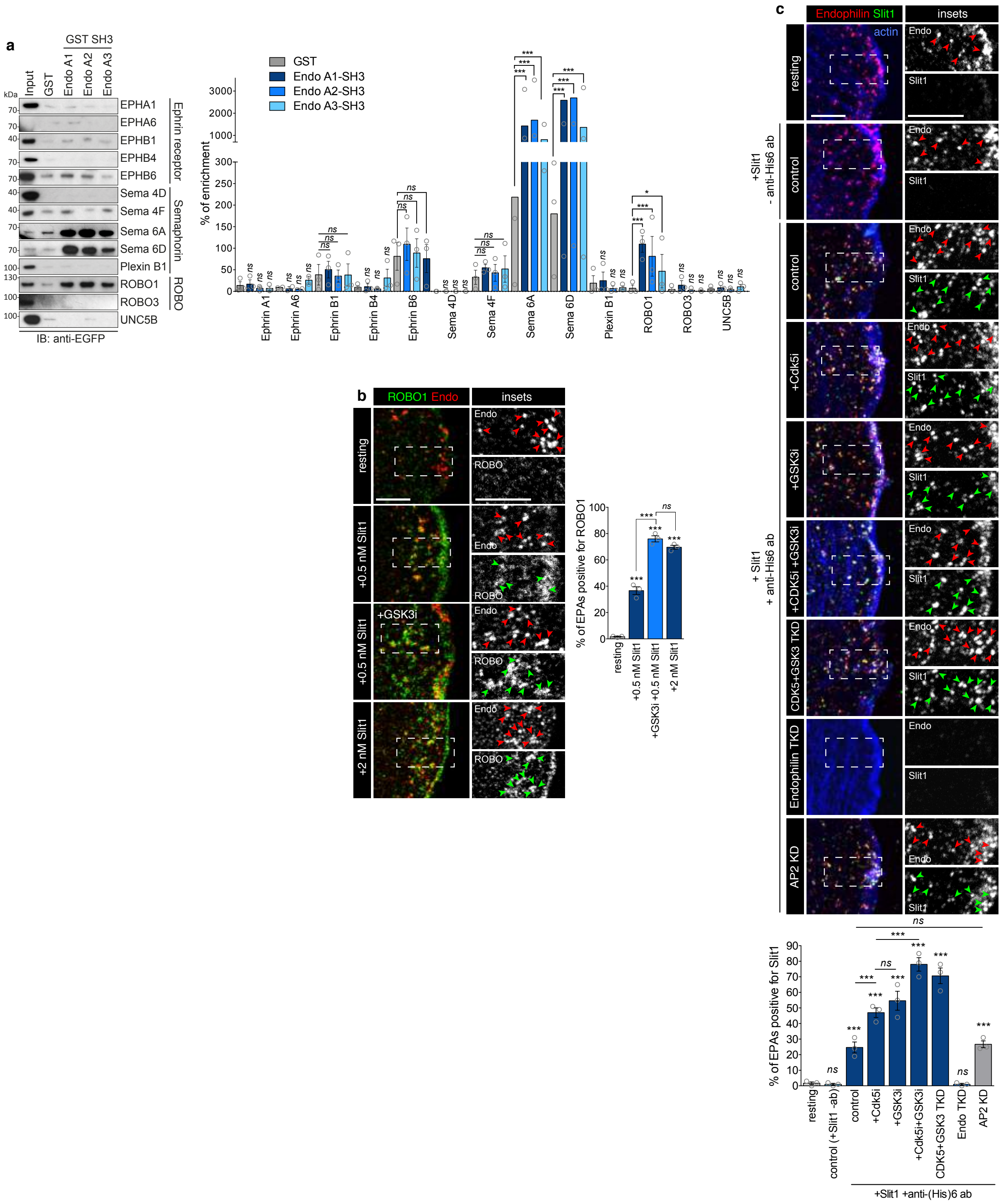

a
GST SH3
Input
GST
Endo A1
Endo A2
Endo A3
kDa
EPHA1
EPHA6
EPHB1
EPHB4
EPHB6
Sema 4D
Sema 4F
Sema 6A
Sema 6D
Plexin B1
ROBO1
ROBO3
UNC5B
Ephrin receptor
Semaphorin
ROBO
IB: anti-EGFP
GST
Endo A1-SH3
Endo A2-SH3
Endo A3-SH3
% of enrichment
Ephrin A1
Ephrin A6
Ephrin B1
Ephrin B4
Ephrin B6
Sema 4D
Sema 4F
Sema 6A
Sema 6D
Plexin B1
ROBO1
ROBO3
UNC5B
b
ROBO1 Endo
insets
resting
+0.5 nM Slit1
+GSK3i
+2 nM Slit1
Endo
ROBO
% of EPAs positive for ROBO1
+GSK3i +0.5 nM Slit1
c
Endophilin Slit1
actin
insets
+Slit1
- anti-His6 ab
+ Slit1
+ anti-His6 ab
control
+Cdk5i
+GSK3i
+CDK5i +GSK3i
CDK5+GSK3 TKD
Endophilin TKD
AP2 KD
Endo
Slit1
% of EPAs positive for Slit1
resting
control (+Slit1 -ab)
+Cdk5i+GSK3i
Endo TKD
+Slit1 +anti-(His)6 ab

Supplementary Figure 4. *(previous page)* **Related to Figure 5. a**, Left, pull-down using GST-SH3 domains of Endophilin A1, A2 or A3 and cell extracts expressing the indicated EGFP-tagged receptor tails. GST was used as negative control. Binding proteins were detected by immunoblotting with an anti-EGFP antibody. Input lanes correspond to 5% of the cell extracts. Right, Histograms show the mean ± SEM from 3 independent biological experiments. Statistical analysis was performed by two-way ANOVA; *NS*, non significant; *, *P*<0.05, ***, *P*<0.001. **b**, Left, recruitment of endogenous ROBO1 into FEME carriers in HUVEC cells treated for 10min with 5μM CHIR-99021 (+GSK3i) or not before stimulation with 0.5 or 2nM Slit1 for 5min. Arrowheads point at FEME carriers. Scale bars, 5μm. Histograms show the mean ± SEM from 3 independent biological experiments (n=50 EPAs per condition). Statistical analysis was performed by one-way ANOVA; *ns*, non significant; ***, *P*<0.001.
c, Slit1-His_6 uptake (2nM for 5min) into FEME carriers in HUVEC cells depleted of Endophilin A1, A2 and A3 (Endophilin TKD), Cdk5 and GSK3α and β (CDK5+GSK3α/β TKD), or AP2 or pre-treated with 5μM Dinaciclib (Cdk5i) and/or CHIR-99021 (GSK3i) for 10min. Arrowheads point at FEME carriers. Scale bars, 5μm. Histograms show the mean ± SEM from 3 independent biological experiments (*n*=15 cells per condition). Statistical analysis was performed by one-way ANOVA; *ns*, non significant; ***, *P*<0.001.

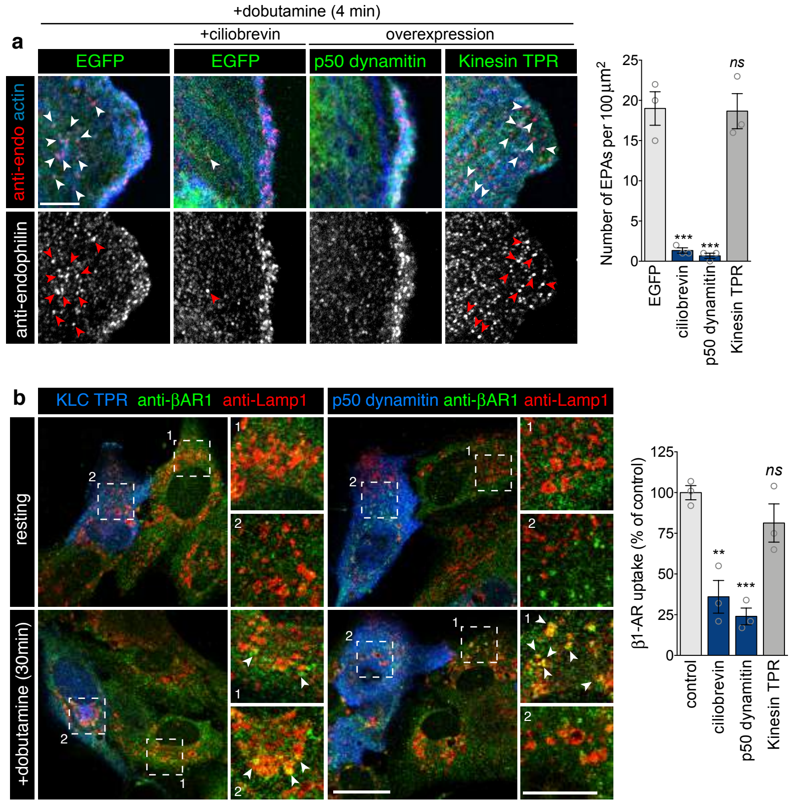

Supplementary Figure 5. Related to Figure 6. a, FEME carrier formation in RPE1 cells treated for 10min with 5μM Ciliobrevin (Dynein inhibitor) or overexpressing EGFP-tagged p50 dynamitin (Dynein dominant-negative) or Kinesin TPR domain (Kinesin dominant-negative), followed by 10μM dobutamine for 4min. EGFP was used as negative control. Arrowheads point at FEME carriers. Scale bar, 5μm. Histograms show the mean ± SEM from 3 independent biological experiments (*n*=20 cells per condition). Statistical analysis was performed by one-way ANOVA; *ns*, non significant; ***, $P<0.001$. **b**, Lysosomal accumulation of β1 adrenergic receptor (β1AR) RPE1 cells overexpressing EGFP-tagged p50 dynamitin or Kinesin TPR domain and treated with 10μM dobutamine for 30min. Arrowheads point at β1AR inside lysosomes. Scale bar, 20μm (main) and 10μm (inset). Histograms show the mean ± SEM from 3 independent biological experiments (*n*=30 cells per condition), normalized to control cells. Statistical analysis was performed by one-way ANOVA; *ns*, non significant; **, $P<0.01$; ***, $P<0.001$.

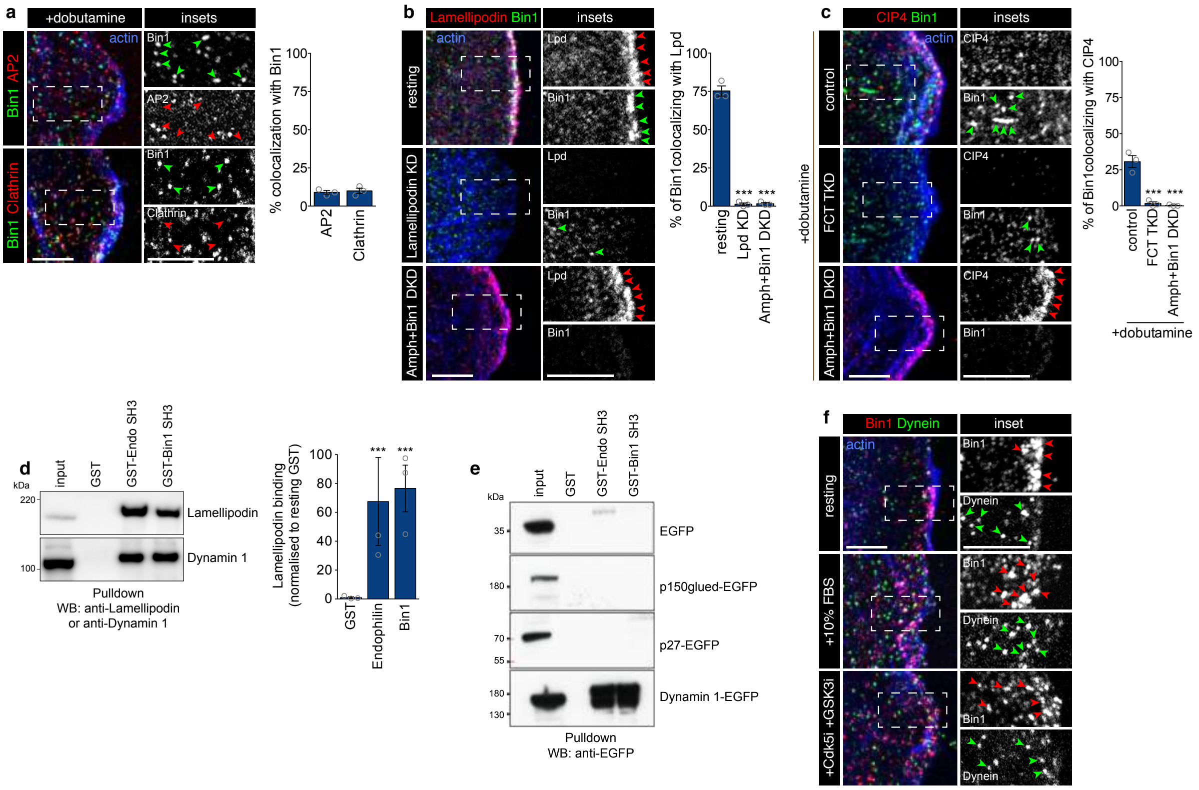

Supplementary Figure 6. Related to Figure 7. **a**, Colocalization of endogenous Bin1 and AP-2 or Clathrin upon stimulation with 10μM dobutamine for 4min. Arrowheads point at Bin1 spots. Scale bars, 5μm. Histograms show the mean ± SEM from 3 independent biological experiments (*n*>150 puncta per staining). **b**, Colocalization of endogenous Bin1 and Lamellipodin in cells depleted of Lamellipodin (Lpd KD), Bin1 Amphiphysin (Amph+Bin1 DKD), or left untreated (resting). Arrowheads point at Bin1 or Lpd spots at the plasma membrane. Scale bars, 5μm. Histograms show the mean ± SEM from 3 independent biological experiments (*n*>150 puncta per condition). Statistical analysis was performed by one-way ANOVA; ***, *P*<0.001. **c**, Colocalization of endogenous Bin1 and CIP4 in cells depleted of FBP17, CIP4 and TOCA-1 (FCT TKD), Bin1 Amphiphysin (Amph+Bin1 DKD), or not (control), and stimulated with 10μM dobutamine for 4min. Scale bars, 5μm. Histograms show the mean ± SEM from 3 independent biological experiments (*n*>150 puncta per condition). Statistical analysis was performed by one-way ANOVA; ***, *P*<0.001. **d**, Pull-down experiments using beads with GST-SH3 domains of Endophilin A2 or Bin1, in resting cells or cells treated with extra 10% FBS for 10min. GST beads were used as negative control. Inputs correspond to 4% of cell extracts. Histograms show the mean ± SEM of Dynein binding, normalized to resting GST levels from 3 independent biological experiments. Statistical analysis was performed by one-way ANOVA; ***, *P*<0.001. **e**, Pull-down experiment using beads with GST only or GST-SH3 domains of endophilin A2 or Bin1. Bound EGFP, p150-glued-EGFP, p27-EGFP or Dynamin 1-EGFP were tested by immunoblot. GST was used as negative control. Input corresponds to 4% of cell extracts. **f**, Related to Figure 7e: whole dataset (note that some images are similar, as Figure 7e shows a subset of the conditions tested). Colocalization between endogenous Bin1 and Dynein in resting cells stimulated with extra 10% serum (middle), or pre-treated with 5μM Dinaciclib (Cdk5i) and/or CHIR-99021 (GSK3i) for 10min before fixation (bottom). Arrowheads points at Bin1-labelled FEME carriers. Scale bars, 5μm.

Supplementary Table 1. Primers used in this study

Constructs	Primers (5’-3’)
human SH3GL1 full-length C-term tag (Forward)	GGGGACAAGTTTGTACAAAAAAGCAGGCTTCGAAGGAGATAGAACCATGGCCTCGGTGGCGGGGCTGAAGAAGCAG
human SH3GL1 full-length C-term tag (Reverse)	GGGGACCACTTTGTACAAGAAAGCTGGGTCCTGCGGCAGGGGCACAAGCACCTC
human SH3GL1 (311-end) N-term tag (Forward)	GGGGACAAGTTTGTACAAAAAAGCAGGCTTGTGCAAGGCGCTGTACGACTTCGAG
human SH3GL1 (311-end) N-term tag (Reverse)	GGGGACCACTTTGTACAAGAAAGCTGGGTCCTACTGCGGCAGGGGCACAAGCACCTCCAC
human SH3GL2 full-length C-term tag (Forward)	GGGGACAAGTTTGTACAAAAAAGCAGGCTTCGAAGGAGATAGAACCATGGCCTCGGTGGCCGGCCTCAAGAAGCAG
human SH3GL2 full-length C-term tag (Reverse)	GGGGACCACTTTGTACAAGAAAGCTGGGTCATGGGGCAGGGCAACCAGAATTTC
human SH3GL2 (295-end) N-term tag (Forward)	GGGGACAAGTTTGTACAAAAAAGCAGGCTTGTGCCGAGCTCTGTACGACTTTGAA
human SH3GL2 (295-end) N-term tag (Reverse)	GGGGACCACTTTGTACAAGAAAGCTGGGTCCTAATGGGGCAGGGCAACCAGAATTTCCAC
human SH3GL3 full-length C-term tag (Forward)	GGGGACAAGTTTGTACAAAAAAGCAGGCTTCGAAGGAGATAGAACCATGGCCTCGGTGGCCGGGCTGAAGAAGCAG
human SH3GL3 full-length C-term tag (Reverse)	GGGGACCACTTTGTACAAGAAAGCTGGGTCCTGAGGTAAAGGCACGATCACTTC
human SH3GL3 (291-end) N-term tag (Forward)	GGGGACAAGTTTGTACAAAAAAGCAGGCTTCGAAGGAGATAGAACCATGGCCCGTGGTCTCTATGACTTTGAGCCA
human SH3GL3 (291-end) N-term tag (Reverse)	GGGGACCACTTTGTACAAGAAAGCTGGGTCCTACTGAGGTAAAGGCACGATCACTTC
human BIN1 full-length C-term tag (Forward)	GGGGACAAGTTTGTACAAAAAAGCAGGCTTCGAAGGAGATAGAACCATGGCAGAGATGGGCAGTAAAGG
human BIN1 full-length C-term tag (Reverse)	GGGGACCACTTTGTACAAGAAAGCTGGGTCTGGGACCCTCTCAGTGAAGTTCTCG
human BIN1 (366-end) N-term tag (Forward)	GGGGACAAGTTTGTACAAAAAAGCAGGCTTGGGTTTCATGTTCAAGGTACAGG
human BIN1 (366-end) N-term tag (Reverse)	GGGGACCACTTTGTACAAGAAAGCTGGGTCCTATGGGACCCTCTCAGTGAAGTTCTC
human DPYSL2 full-length N-term tag (Forward)	GGGGACAAGTTTGTACAAAAAAGCAGGCTTGGCCGAGAGAAAGCAATCCGGGAAG
human DPYSL2 full-length N-term tag (Reverse)	GGGGACCACTTTGTACAAGAAAGCTGGGTCCTACTAGCCCAGGCTGGTGATGTTGG
mouse DPYSL3 full-length N-term tag (Forward)	GGGGACAAGTTTGTACAAAAAAGCAGGCTTGGCCTCGGGCCGGAGGG
mouse DPYSL3 full-length N-term tag (Reverse)	GGGGACCACTTTGTACAAGAAAGCTGGGTCCTAACTCAGAGATGTGATATTAGAACG
mouse DPYSL3 P502A (Forward)	AGA GGC ATG TAT GAT GGA GCA GTG TTT GAC TTG ACC ACC
mouse DPYSL3 P502A (Reverse)	GGT GGT CAA GTC AAA CAC TGC TCC ATC ATA CAT GCC TCT
mouse DPYSL3 S522A (Forward)	GCT GGC TCT ACT CGG GGC GCT CCC ACT CGG CCA AAC CC
mouse DPYSL3 S522A (Reverse)	GGG TTT GGC CGA GTG GGA GCG CCC CGA GTA GAG CCA GC
mouse DPYSL3 S522D (Forward)	GCT GGC TCT ACT CGG GGC GAT CCC ACT CGG CCA AAC CC
mouse DPYSL3 S522D (Reverse)	GGG TTT GGC CGA GTG GGA TCG CCC CGA GTA GAG CCA GC
mouse DPYSL3 R525E (Forward)	CTCGG GGC TCT CCC ACT GAG CCA AAC CCG CCA GTG AGG
mouse DPYSL3 R525E (Reverse)	CCT CAC TGG CGG GTT TGG CTC AGT GGG AGA GCC CCGAG
mouse DPYSL3 P526A (Forward)	GGG CTC TCC CAC TCG GGC AAA CCC GCC AGT GAG G
mouse DPYSL3 P526A (Reverse)	CCT CAC TGG CGG GTT TGC CCG AGT GGG AGA GCC C
human DPYSL5 full-length N-term tag (Forward)	GGGGACAAGTTTGTACAAAAAAGCAGGCTTGCTTGCCAACTCAGCCAGCG
human DPYSL5 full-length N-term tag (Reverse)	GGGGACCACTTTGTACAAGAAAGCTGGGTCCTACCAAATGCCACTCGACCTGCC
human EPHA1 (568-976) N-term tag (Forward)	GGGGACAAGTTTGTACAAAAAAGCAGGCTTGCGGTCCAGGAGAGCCCAG
human EPHA1 (568-976) N-term tag (Reverse)	GGGGACCACTTTGTACAAGAAAGCTGGGTCCTA GTCCTTGAATCCCTGAATACTGC
human EPHA6 (571-1035) N-term tag (Forward)	GGGGACAAGTTTGTACAAAAAA GCA GGC TTC ACC ATG GCCACTGGGAGATGTCAGTGGTA
human EPHA6 (571-1035) N-term tag (Reverse)	GGGGACCACTTTGTACAAGAAAGCTGGGTCTACATGAAATCCCTTCTCCTGTA
human EPHB1 (259-976) N-term tag (Forward)	GGGGACAAGTTTGTACAAAAAAGCAGGCTTGAGCAGGAAACGGGCTTATAGC
human EPHB1 (259-976) N-term tag (Reverse)	GGGGACCACTTTGTACAAGAAAGCTGGGTCCTATGCCATTGCCGTTGGTG
human EPHB4 (561-987) N-term tag (Forward)	GGGGACAAGTTTGTACAAAAAAGCAGGCTTG CTCTGCCTCAGGAAGCAGAGC
human EPHB4 (561-987) N-term tag (Reverse)	GGGGACCACTTTGTACAAGAAAGCTGGGTCCTA GTACTGCGGGGCCGGTC
human EPHB6 (616-1021) N-term tag (Forward)	GGGGACAAGTTTGTACAAAAAAGCAGGCTTG GCGGTCGTCTTCCAGCG
human EPHB6 (616-1021) N-term tag (Reverse)	GGGGACCACTTTGTACAAGAAAGCTGGGTCCTA GACCTCCACTGAGCCCTG
human SEMA4D (756-862) N-term tag (Forward)	GGGGACAAGTTTGTACAAAAAAGCAGGCTTGTATAAGGGATACCTGCCCAG
human SEMA4D (756-862) N-term tag (Reverse)	GGGGACCACTTTGTACAAGAAAGCTGGGTCCTAGTCTCCATCTGCGTCTGAG
human SEMA4F (681-770) N-term tag (Forward)	GGGGACAAGTTTGTACAAAAAAGCAGGCTTGGGTCGGCGTCAGCAGCGACG
human SEMA4F (681-770) N-term tag (Reverse)	GGGGACCACTTTGTACAAGAAAGCTGGGTCCTAGATGGATGTTTCATCACATGTGG
human SEMA6A (671-1030) N-term tag (Forward)	GGGGACAAGTTTGTACAAAAAAGCAGGCTTGTACTGCGTCTGTGATCATCG
human SEMA6A (671-1030) N-term tag (Reverse)	GGGGACCACTTTGTACAAGAAAGCTGGGTCCTATGTACACGCATCATTGGGCTTC
human SEMA6D (684-1073) N-term tag (Forward)	GGGGACAAGTTTGTACAAAAAAGCAGGCTTGTACTGCTATCGAGACATGTTTGTTCG
human SEMA6D (684-1073) N-term tag (Reverse)	GGGGACCACTTTGTACAAGAAAGCTGGGTCCTAGTATGTGTATTTGTTCAGTGG
human PLXNB1 (1512-2135) N-term tag (Forward)	GGGGACAAGTTTGTACAAAAAAGCAGGCTTGAGGAGGAAGAGCAAGCAGGC
human PLXNB1 (1512-2135) N-term tag (Rev)	GGGGACCACTTTGTACAAGAAAGCTGGGTCCTATAGATCTGTGACCTTGTTTTCC
human ROBO1 (919-1651) N-term tag (Forward)	GGGGACAAGTTTGTACAAAAAAGCAGGCTTG TATCGACACCGCAAGAAG
human ROBO1 (919-1651) N-term tag (Reverse)	GGGGACCACTTTGTACAAGAAAGCTGGGTCCTA GCTTTCAGTTTCCTCTAATTC
human ROBO3 (913-1386) N-term tag (Forward)	GGGGACAAGTTTGTACAAAAAAGCAGGCTTG TACTGGCGCCGGAAACAG
human ROBO3 (913-1386) N-term tag (Reverse)	GGGGACCACTTTGTACAAGAAAGCTGGGTCCTA TCTTGGTTCCTCTCGGCG
human UNC5B (399-945) N-term tag (Forward)	GGGGACAAGTTTGTACAAAAAAGCAGGCTTCACCATG GCCTACCGCCGCAACTGCCGTG
human UNC5B (399-945) N-term tag (Reverse)	GGGGACCACTTTGTACAAGAAAGCTGGGTC GCAGTCCCCGTCGGTGGC
rat DNM1 S774A (Forward)	GGA CGC AGG GCG CCC ACG TCC
rat DNM1 S774A (Reverse)	GGA CGT GGG CGC CCT GCG TCC
rat DNM1 S778A (Forward)	CCC ACG TCC GCC CCC ACG CCG
rat DNM1 S778A (Reverse)	CGG CGT GGG GGC GGA CGT GGG
