# Supplementary material for: Cdk5 and GSK3β inhibit fast endophilin-mediated endocytosis
Source: Nat Commun. 2021 Apr 23;12:2424. doi: 10.1038/s41467-021-22603-4 (PMC8065113; doi:10.1038/s41467-021-22603-4)
Supplement: Supplementary file 3 — Reporting Summary [file 41467_2021_22603_MOESM3_ESM.pdf]

nature research

Corresponding author(s): Emmanuel Boucrot
Last updated by author(s): 09/02/2021

Reporting Summary

Nature Research wishes to improve the reproducibility of the work that we publish. This form provides structure for consistency and transparency in reporting. For further information on Nature Research policies, see our Editorial Policies and the Editorial Policy Checklist.

Statistics

For all statistical analyses, confirm that the following items are present in the figure legend, table legend, main text, or Methods section.

n/a	Confirmed	
☐	☒	The exact sample size (*n*) for each experimental group/condition, given as a discrete number and unit of measurement
☐	☒	A statement on whether measurements were taken from distinct samples or whether the same sample was measured repeatedly
☐	☒	The statistical test(s) used AND whether they are one- or two-sided<br>*Only common tests should be described solely by name; describe more complex techniques in the Methods section.*
☒	☐	A description of all covariates tested
☐	☒	A description of any assumptions or corrections, such as tests of normality and adjustment for multiple comparisons
☐	☒	A full description of the statistical parameters including central tendency (e.g. means) or other basic estimates (e.g. regression coefficient) AND variation (e.g. standard deviation) or associated estimates of uncertainty (e.g. confidence intervals)
☐	☒	For null hypothesis testing, the test statistic (e.g. *F*, *t*, *r*) with confidence intervals, effect sizes, degrees of freedom and *P* value noted<br>*Give P values as exact values whenever suitable.*
☒	☐	For Bayesian analysis, information on the choice of priors and Markov chain Monte Carlo settings
☒	☐	For hierarchical and complex designs, identification of the appropriate level for tests and full reporting of outcomes
☒	☐	Estimates of effect sizes (e.g. Cohen's *d*, Pearson's *r*), indicating how they were calculated

Our web collection on statistics for biologists contains articles on many of the points above.

Software and code

Policy information about availability of computer code

Data collection	Volocity 6.0 (Improvision, Perkin Elmer), LAS X (Leica), Image Lab 6.1 (Bio-Rad), Progenesis v4.0 (Waters, UK).
Data analysis	Excel 14.7.3 (Microsoft), ImageJ 1.51s (NIH), Prism 6 (GraphPad), Photoshop CS5 (Adobe), Image Lab 6.1 (Bio-Rad)

For manuscripts utilizing custom algorithms or software that are central to the research but not yet described in published literature, software must be made available to editors and reviewers. We strongly encourage code deposition in a community repository (e.g. GitHub). See the Nature Research guidelines for submitting code & software for further information.

Data

Policy information about availability of data

All manuscripts must include a data availability statement. This statement should provide the following information, where applicable:
- Accession codes, unique identifiers, or web links for publicly available datasets
- A list of figures that have associated raw data
- A description of any restrictions on data availability

The source data for statistical analysis of all the measurements used in this study is provided in the Source Data File. LC-MS/MS data have been deposited in ProteomeXchange Consortium via the PRIDE partner repository with identifier PXD021138. All uncropped gels and blots are provided in the Source Data File. All other data that support the findings of this study are available from the corresponding author upon reasonable request.

Field-specific reporting

Please select the one below that is the best fit for your research. If you are not sure, read the appropriate sections before making your selection.

- [x] Life sciences
- [ ] Behavioural & social sciences
- [ ] Ecological, evolutionary & environmental sciences

For a reference copy of the document with all sections, see nature.com/documents/nr-reporting-summary-flat.pdf

Life sciences study design

All studies must disclose on these points even when the disclosure is negative.

Sample size	The samples sizes and statistical tests were selected based on previous studies with similar methodologies (Boucrot et al. 2015 Nature 517 (7535):460-5 and Chan Wah Hak et al. 2018 Nat Cel Biol 20(9):1023-31).<br>The sample sizes were sufficient to yield hight statistical significance.
Data exclusions	No data was excluded from analysis.
Replication	All experiments were repeated at least three times, giving similar results.
Randomization	No randomization was used based on previous studies with similar methodologies.
Blinding	No blinding was used based on previous studies with similar methodologies.

Reporting for specific materials, systems and methods

We require information from authors about some types of materials, experimental systems and methods used in many studies. Here, indicate whether each material, system or method listed is relevant to your study. If you are not sure if a list item applies to your research, read the appropriate section before selecting a response.

Materials & experimental systems

n/a	Involved in the study	
☐	☒	Antibodies
☐	☒	Eukaryotic cell lines
☒	☐	Palaeontology and archaeology
☒	☐	Animals and other organisms
☒	☐	Human research participants
☒	☐	Clinical data
☒	☐	Dual use research of concern

Methods

n/a	Involved in the study	
☒	☐	ChIP-seq
☒	☐	Flow cytometry
☒	☐	MRI-based neuroimaging

Antibodies

Antibodies used	The following primary antibodies were used for immunostaining or immunoblotting: anti-EGFP ab290 (rabbit polyclonal, AbCam290) used at 0.5µg/mL (1:8,000 dilution), anti-EGFP clones 7.1 and 13.1 (mouse monoclonal, Roche 11814460001) used at 0.5µg/mL (1:1,000 dilution), anti-Endophilin A2 clone H-60 (rabbit polyclonal, Santa Cruz 25495)), used at 1µg/mL (1:100 dilution), anti-Endophilin A2 clone A-11 (mouse polyclonal, Santa Cruz 365704) used at 1µg/mL (1:200 dilution), anti-β1 adrenergic receptor (rabbit polyclonal, AbCam ab3442) used at 1µg/mL (1:1,000 dilution), anti-CRMP4 (rabbit polyclonal, Milipore 5454) used at 2µg/mL (1:100 dilution), anti-Dynein clone 74.1 (mouse monoclonal, eBioscience 14-9772-80) used at 2µg/mL (1:100 dilution), anti-Plexin A1 (rabbit polyclonal recognizing the ectodomain of PlexinA1, AbCam Ab32960) used at 1µg/mL (1:100 dilution), anti-ROBO1 (sheep polyclonal, AF7118 R&D Systems) used at 1µg/mL (1:500 dilution), anti-LAMP-1 (mouse monoclonal clone H4A3-c, Developmental Studies Hybridoma Bank) used at 1:5,000 dilution, anti-phosphorylated Ser9 GSK3β clone D85E12 (rabbit monoclonal, Cell Signaling Technology 5558) used at 1µg/mL (1:400 dilution), anti-GSKα/β D75D3 (rabbit polyclonal, Cell Signaling Technology 5676) used at 1µg/mL (1:400 dilution), anti-Dynamin 1 clone 41 (mouse monoclonal, BD Pharmigen 610245) used at 1µg/mL (1:250 dilution), anti-Bin1 (rabbit polyclonal, GeneTex GTX103259) used at 2µg/mL (1:500 dilution), anti-Lamellipodin (rabbit polyclonal, Atlas Antibodies HPA020027) used at 2µg/mL (1:50 dilution), anti-CIP4, (mouse monoclonal clone 21, Santa Cruz sc-135868) used at 1µg/mL (1:200 dilution), anti-α Tubulin clone TUB2.1 (mouse monoclonal, AbCam ab11308) used at 0.5µg/mL (1:2,000 dilution), and anti-His tag clone D3I10 (rabbit polyclonal, Cell Signaling Technology 12698) used at 1µg/mL (1:400 dilution). The following secondary antibodies were used for microscopy: Alexa Fluor 488 goat anti-mouse IgG (Thermo Scientific A-11001), Alexa Fluor 555 goat anti-mouse IgG, (Thermo Scientific A-21422), Alexa Fluor 488 goat anti-rabbit IgG, (Thermo Scientific A-11008), Alexa Fluor 555 goat anti-rabbit IgG, (Thermo Scientific A-21428), Alexa Fluor 388 donkey anti-Sheep IgG (Thermo Scientific A-11015), Alexa Fluor 555 donkey anti-mouse IgG (Thermo Scientific A-31570), and for immunoblot; goat anti-mouse IgG-HRP conjugated (Bio-Rad 1706516) and goat anti-rabbit IgG-HRP conjugated (Bio-Rad 1706519)..
Validation	anti-EGFP ab290 (rabbit polyclonal, AbCam290) was validated by western-blotting and immunofluorescence on overexpressing samples in Boucrot et al. 2015 (PMID: 25517094); anti-EGFP clones 7.1 and 13.1 (mouse monoclonal, Roche 11814460001) was

validated by western-blotting and immunofluorescence on overexpressing samples in Boucrot et al. 2015 (PMID: 25517094); anti-Endophilin A2 clone H-60 (rabbit polyclonal, Santa Cruz 25495) was validated by western-blotting and immunofluorescence on RNAi-treated samples in Boucrot et al. 2015 (PMID: 25517094); anti-Endophilin A2 clone A-11 (mouse polyclonal, Santa Cruz 365704) was validated by western-blotting and immunofluorescence on RNAi-treated samples in Boucrot et al. 2015 (PMID: 25517094); anti-β1 adrenergic receptor (rabbit polyclonal, AbCam ab3442) was validated by immunofluorescence on RNAi-treated samples in Irannejad et al. 2017 (PMID: 28553949); anti-CRMP4 (rabbit polyclonal, Milipore 5454) was validated by western-blotting and immunofluorescence on KO samples in Nagai et al. 2015 (PMID: 25652774); anti-Dynein clone 74.1 (mouse monoclonal, eBioscience 14-9772-80) was validated by western-blotting and immunofluorescence in Nübe et al 2016 (PMID: 26845719); anti-Plexin A1 (rabbit polyclonal recognizing the ectodomain of PlexinA1, AbCam Ab32960) was validated by western-blotting and immunofluorescence on KO samples in Delloye-Bourgeois et al. 2015 (PMID: 25485759); anti-ROBO1 (sheep polyclonal, AF7118 R&D Systems) was validated by neutralization in Pilling et al. 2018 (PMID: 30510066); anti-LAMP-1 (mouse monoclonal clone H4A3-c, Developmental Studies Hybridoma Bank) was validated by western-blotting and immunofluorescence on RNAi-treated samples in Krewski et al. 2013 (PMID: 23632890); anti-phosphorylated Ser9 GSK3β clone D85E12 (rabbit monoclonal, Cell Signaling Technology 5558) was validated by western-blotting by Dr Jim Woodgett (U. of Toronto, Canada), as displayed on the Cell Signaling Technology webpage for the antibody; anti-GSKα/β D75D3 (rabbit polyclonal, Cell Signaling Technology 5676) was validated by western-blotting by Dr Jim Woodgett (U. of Toronto, Canada), as displayed on the Cell Signaling Technology webpage for the antibody; anti-Dynamin 1 clone 41 (mouse monoclonal, BD Pharmigen 610245) was validated by western-blotting and immunofluorescence on KO samples in Soda et al. 2012 (PMID: 23187129); anti-Bin1 (rabbit polyclonal, GeneTex GTX103259) was validated by immunofluorescence on RNAi-treated samples in Chan Wah Hak et al. 2018 (PMID: 30061681); anti-Lamellipodin (rabbit polyclonal, Atlas Antibodies HPA020027) was validated by immunofluorescence on RNAi-treated samples in Chan Wah Hak et al. 2018 (PMID: 30061681); anti-CIP4, (mouse monoclonal clone 21, Santa Cruz sc-135868) was validated by immunofluorescence on RNAi-treated samples in Chan Wah Hak et al. 2018 (PMID: 30061681); anti-αTubulin clone TUB2.1 (mouse monoclonal, AbCam ab11308) was validated by immunofluorescence on nocodazole-treated samples in Wobser al. 2019 (PMID: 30885250) and anti-His tag clone D3I10 (rabbit polyclonal, Cell Signaling Technology 12698) was validated by immunofluorescence on overexpressing samples in Mo al. 2016 (PMID: 26829474).

Eukaryotic cell lines

Policy information about cell lines

Cell line source(s)	hTERT-RPE1 from ATCC (CRL-4000), Human Primary Dermal Fibroblasts from ATCC (PCS-201-012), Human Umbilical Vein Endothelial Cells HUVEC from (PCS-100-010), BSC-1 from ECAAC (85011422), HeLa from ATCC (CCL-2) and HEK293 from ATCC (CRL-1573)
Authentication	BSC-1 were authenticated by DNA barcoding by the provider (ECAAC), HEK293, HeLa and hTERT-RPE1 were authenticated by STR profile by the provider (ATCC). Human Primary Dermal Fibroblasts and Human Umbilical Vein Endothelial Cells are primary cells from healthy donors.
Mycoplasma contamination	Cells tested negative for mycoplasma contamination at the time of the experiments (PCR test)
Commonly misidentified lines (See ICLAC register)	no commonly misidentified lines
